# Merkel cell polyomavirus (MCV) T-antigen seroreactivity, MCV DNA in eyebrow hairs, and squamous cell carcinoma

**DOI:** 10.1186/s13027-015-0030-0

**Published:** 2015-10-19

**Authors:** Shalaka S. Hampras, Angelika Michel, Markus Schmitt, Tim Waterboer, Lena Kranz, Tarik Gheit, Kate Fisher, Vernon K. Sondak, Jane Messina, Neil Fenske, Basil Cherpelis, Massimo Tommasino, Michael Pawlita, Dana E. Rollison

**Affiliations:** Department of Cancer Epidemiology, Moffitt Cancer Center, Tampa, FL 33612 USA; Infection and Cancer Program, German Cancer Research Center, Heidelberg, Germany; Present address: GATC Biotech AG, Constance, Germany; Present address: Research Center for Immunotherapy (FZI), Langenbeckstrasse 1, Building 708, 55131 Mainz, Germany; Infections and Cancer Biology Group, International Agency for Research on Cancer-World Health Organization, Lyon, 69372 France; Department of Biostatistics and Bioinformatics, Moffitt Cancer Center, Tampa, FL USA; Cutaneous Oncology Program, Moffitt Cancer Center, Tampa, FL USA; Departments of Pathology and Cell Biology, University of South Florida College of Medicine, Tampa, FL USA; Dermatology, University of South Florida College of Medicine, Tampa, FL USA; Cutaneous Surgery, University of South Florida College of Medicine, Tampa, FL USA

**Keywords:** Merkel cell polyomavirus, Cutaneous squamous cell carcinoma, T-Antigen, Eyebrow hairs

## Abstract

**Background:**

The role of Merkel cell polyomavirus (MCV) infection in the etiology of non-melanoma skin cancers, other than Merkel cell carcinoma, is unclear. Previously, we reported a significant association between seropositivity to MCV capsid antigen and MCV DNA-positive cutaneous squamous cell carcinoma (SCC). Here we present associations between SCC and seroreactivity to MCV T-antigen (T-Ag) oncoprotein, as well as MCV DNA detected in eyebrow hairs.

**Findings:**

A clinic-based case–control study, including 171 SCC cases and 300 controls without skin cancer, was conducted at Moffitt Cancer Center in Tampa, Florida. Multiplex assays were used to measure serum antibodies against MCV small and large T-Ag and MCV DNA in both eyebrow hairs and SCC tumors (*n* = 144). Odds ratios (ORs) and 95 % confidence intervals (CIs) were estimated using logistic regression to evaluate the associations between MCV and SCC. No significant association was observed between seroreactivity to MCV full-length large or small T-Ag and SCC, overall [OR_large T-Ag_ = 0.99 (0.48-2.08), OR_small T-Ag_ = 0.31 (0.06–1.62)] or when comparing tumor MCV DNA-positive cases to controls [OR_large T-Ag_ = 1.06 (0.38–2.93)]. Only presence of MCV DNA in eyebrow hairs was significantly associated with MCV DNA-positive SCC [OR = 4.05 (2.01–8.18)].

**Conclusion:**

MCV infection is unlikely to play a direct role in SCC.

## Findings

### Introduction

While Merkel cell polyomavirus (MCV) DNA has been consistently detected in more than 80 % of Merkel cell carcinoma (MCC) [1–3], the role of MCV in the development of other non-melanoma skin cancers, such as cutaneous squamous cell carcinoma (SCC), is not established. Previously, two studies reported presence of MCV DNA in 0 % of SCC tumors [4, 5], while six other studies showed MCV DNA in 13–38 % of SCC tumors [6–11]. The one study that examined oncoprotein expression [4] reported no MCV T-antigen (T-Ag) expression in secondary SCCs detected among MCC patients.

In our previous case–control study, MCV DNA was detected in 38 % of 145 SCC tumor tissues and significant association between seropositivity to MCV capsid antigen (VP1) and MCV DNA-positive SCC was observed [11]. However, given the high seroprevalence of viral capsid proteins in cancer free individuals [12], seropositivity to MCV capsid antigen may not reflect oncogenic viral infection and additional biomarkers of MCV infection should be explored.

Truncating mutations in MCV large T-Ag C-terminus are thought to promote tumor cell proliferation [13]. While the oncogenic role of MCV T-Ag has been established in MCC [13, 14], SCC tumors lack the tumor promoting mutations in large T-Ag observed in MCC [7]. Previous studies examining the role of MCV in SCC were limited by small sample size and only examined either MCV large T-Ag or VP1 protein sequence/expression in SCC [4, 7, 8, 6, 9]. We expanded our previous case–control analysis [11] to examine seroreactivity to both MCV large T-Ag and small T-Ag in association with SCC. Since serological response to MCV antigens may represent current and past infection and can possibly be affected by host factors associated with immune response, we also examined the association between MCV DNA present in eyebrow hairs and SCC.

## Materials and methods

### Study population

The study population has been described in detail previously [11]. Briefly, a clinic-based case–control study was conducted at Moffitt Cancer Center, Tampa, Florida in 2007–2009. Cases included newly diagnosed, histologically confirmed cases of SCC, a majority of which were immunocompetent [11], and were identified through the University of South Florida (USF) Dermatology Clinic. Controls included patients without history of cancer and had a negative skin cancer screening exam at the USF Family Medicine Clinic or Moffitt’s cancer screening clinic. Of the 173 SCC cases and 300 controls included in the MCV capsid antibody analysis [11], MCV T-Ag serology results were available for 171 SCC cases and 300 controls. Eyebrow hair samples were available for 169 SCC cases and 292 controls. All study participants completed a comprehensive questionnaire on demographics, lifestyle and skin cancer risk factors.

### Ethics, consent and permissions

All participants provided written informed consent, and the study protocol was approved by the Institutional Review Board at USF.

### MCV serology

Serum antibodies (IgG) to small T-Ag, full-length large T-Ag, large T-Ag exon 1 and large T-Ag exon 2 of MCV (isolate 344) were measured using a fluorescent bead-based multiplex assay, as described previously [15, 11, 16]. Briefly, seroreactivity to MCV T-Ag was expressed as median fluorescence intensity (MFI). The cut-offs for seropositivity were determined using an independent reference sample of 42 MCC patients (200 MFI for small T-Ag, 200 MFI for large T-Ag exon 1,400 MFI for large T-Ag exon 2 and 400 MFI for full-length large T-Ag).

### MCV DNA measurement in SCC tumor tissues

As described previously, MCV DNA from eyebrow hairs and fresh frozen SCC tumor tissue was detected using a highly sensitive and specific assay which combines multiplex polymerase chain reaction (PCR) and bead-based Luminex technology [11, 17]. MCV viral load (absolute copy number per sample) in SCC tumor tissue was determined using a multiplex quantitative real-time PCR targeting the N-terminus and C-terminus of the MCV T-antigen sequence [18]. The ratio of N-terminus to C-terminus copy numbers of MCV DNA was determined to examine the presence of C-terminus deletions. Two samples of formalin fixed paraffin embedded MCC tumor tissues were analyzed as positive controls. Data on both MCV T-Ag serology and MCV tumor DNA were available from 144 SCC cases, while data on MCV DNA in eyebrow hair and MCV DNA in SCC tumor were available from 141 SCC cases.

### Statistical analysis

Associations between MCV T-Ag seropositivity, MCV DNA in eyebrow hairs and SCC were estimated by odds ratios (OR) and 95 % confidence intervals (CI) calculated using logistic regression with adjustment for age and gender. Odds ratios were calculated separately for MCV DNA-positive SCC cases and MCV DNA-negative SCC cases using multinomial logistic regression, also adjusting for age and gender. Analyses were conducted using SAS software, version 9.3 (SAS institute Inc., Cary, North Carolina) and R software, version 2.15.1 [19].

## Results

Demographic characteristics of the study population and factors associated with SCC have been described previously [11]. Cases (mean age = 64.4, standard deviation = 9.9) were significantly older compared to controls (mean age = 55.4, standard deviation = 11.7, p value = <0.0001), and were more likely to be males (65.9 %) compared to controls (38.0 %, P value = <0.0001) [11]. As seen in Table 1, 0.3–1 % of controls and 0–1.2 % of SCC cases were seropositive to MCV T-Ags based on MFI cut-offs determined using a reference sample of MCC cases. Due to the small number of seropositive cases and controls, robust statistical analyses to examine age-adjusted association of MCV seropositivity with SCC could not be conducted. Hence, a less stringent cut-off of >50 MFI for seropositivity was used and no significant associations between seropositivity to any of the MCV antigens and SCC were observed, after adjusting for age and gender (Table 1). MCV DNA was present in eyebrow hairs for 37.3 % of controls and 48.5 % of SCC cases, a difference that was not statistically significant (Table 1).

**Table 1:** Associations between Merkel cell polyomavirus and cutaneous squamous cell carcinoma

MCV biomarker	Controls	SCC Cases	Controls	SCC Cases	OR (95 % CI)	*p*-value
	*N* = 300	*N* = 171	*N* = 300	*N* = 171		
	n (%)	n (%)	n (%)	n (%)		
Seropositivity to MCV T-Ag	Cut points based on reference sample*		Cut point of > 50 MFI			
MCV small T	1 (0.3)	0 (0)	7 (2.3)	2 (1.2)	0.31 (0.06–1.62)	0.164
MCV large T exon 1	2 (0.7)	0 (0)	3 (1)	1 (0.6)	0.56 (0.05–5.93)	0.633
MCV large T exon 2	2 (0.7)	1 (0.6)	18 (6)	16 (9.4)	1.22 (0.57–2.62)	0.611
MCV large T	3 (1.0)	2 (1.2)	22 (7.3)	16 (9.4)	0.99 (0.48–2.08)	0.988
			Controls (*n* = 292)	Cases (*n* = 169)	OR (95 % CI)	*p*-value
			n(%)	n(%)		
Presence of MCV DNA in eyebrow hairs			109 (37.3)	82 (48.5)	1.17 (0.76–1.78)	0.478

*MCV* Merkel cell polyomavirus, *SCC* cutaneous squamous cell carcinoma. *MFI cutoff for seropositivity based on MCC patients: 200 for small T, 200 for large T exon 1,400 for large T exon 2 and 400 for large T. Odds ratios (OR) and Wald 95 % confidence intervals (CI) calculated using alternative cutoff of >50 MFI and logistic regression, adjusting for age and gender

When analyses were stratified by the presence of MCV DNA in SCC tumors, no associations were observed with seroreactivity to MCV large T-Ag (Table 2). A statistically significant four-fold association was observed between the presence of MCV DNA in eyebrow hairs and MCV DNA-positive SCC versus controls (OR = 4.05, 95 % CI = 2.01–8.18), while no association was observed with MCV DNA-negative SCC versus controls (OR = 0.58, 95 % CI = 0.34–1.01) (Table 2).

**Table 2:** Associations between Merkel cell polyomavirus and cutaneous squamous cell carcinoma by tumor MCV DNA status

	Controls (*N* = 300)	MCV tumor-negative SCC cases (*N* = 89)		MCV tumor-positive SCC cases (*N* = 55)		*p*-value
MCV biomarker	n (%)	n (%)	OR (95 % CI)*	n (%)	OR (95 % CI)*	
Seropositivity to MCV T-Ag (>50 MFI)						
MCV small T-Ag	7 (2.3)	2 (2.2)	0.63 (0.12–3.32)	0 (0)	Could not be estimated	0.862
MCV large T-Ag exon 1	3 (1.0)	1 (1.1)	1.11 (0.11–11.61)	0 (0)	Could not be estimated	0.996
MCV large T-Ag exon 2	18 (6)	10 (11.2)	1.50 (0.63–3.53)	5 (9.1)	1.06 (0.35–3.2)	0.640
MCV large T-Ag	22 (7.3)	9 (10.1)	1.10 (0.47–2.6)	6 (10.9)	1.06 (0.38–2.93)	0.975
	Controls	MCV-DNA negative SCC cases		MCV-DNA positive SCC cases		p-value
	(*n* = 292)	(*n* = 86)		(*n* = 55)		
		n (%)	OR (95 % CI)*	n (%)	OR (95 % CI)*	
MCV DNA present in eyebrow hairs	109 (37.3)	26 (30.2)	0.58 (0.34–1.01)	42 (76.4)	4.05 (2.01–8.18)	1.06 × 10 ^−6^

*MCV* Merkel cell polyomavirus, *SCC* cutaneous squamous cell carcinoma. * Odds ratios (OR) and 95 % confidence intervals (CI) calculated separately SCC cases with or without MCV DNA in their tumors using polynomial logistic regression, adjusting for age and gender

**Fig. 1:**
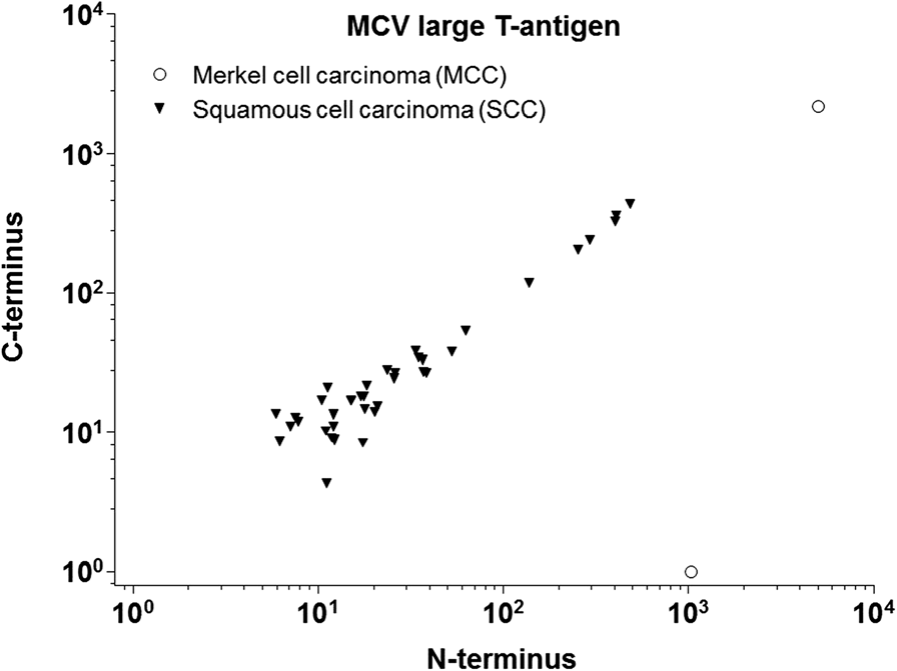
Merkel cell polyomavirus DNA load in squamous cell carcinoma and merkel cell carcinoma tumor tissues. Absolute copy numbers of MCV DNA (N-terminus (X-axis) and C-terminus (Y-axis) sequence) per sample of cutaneous squamous cell carcinoma (filled triangles) detected using multiplex qPCR (without beta globin) are shown. MCV DNA copy numbers in two merkel cell carcinoma samples (empty circles) are also shown. Overall, MCV DNA copy numbers were lower in squamous cell carcinoma samples than in merkel cell carcinoma. There was no indication of C-terminal deletion of T-antigen in MCV detected in squamous cell carcinoma samples

Overall, MCV copy numbers were lower for SCC compared to the two MCC tumor samples analyzed (Fig. 1). The ratio of C- to N-terminus of all SCC samples was close to 1.0, indicating the presence of full-length large T-Ag.

## Discussion

In this clinic based case–control study, no significant association was observed between seropositivity to MCV T-Ag and SCC, overall or after stratifying by MCV DNA status in SCC tumor tissues. Interestingly, a greater than four-fold significant association was observed between MCV DNA positivity in eyebrow hairs and MCV DNA positive-SCC, although it should be noted that MCV viral DNA load was low in SCC tumors, with all exhibiting less than one viral copy per tumor cell.

Previously, in the same study population, we observed a greater than two-fold association between MCV DNA-positive SCC and seropositivity to the MCV capsid antigen, VP1 [11]. However, in our previous study [11], 73.3 % controls and 80.9 % SCC cases were seropositive for VP1. In contrast, we report here <2 % of cases and controls were seropositive for MCV large T-Ag using the cut-off derived from MCC patients. As discussed previously [16], unlike viral capsid proteins, MCV T-Ags are located within the nucleus and are less likely to stimulate a serological response unless the T-Ag is expressed in tumors. While T-Ag DNA sequences have been detected in MCV-positive SCC tumor tissues [6], their expression has not been observed in SCC tumor tissues arising in MCC patients [4] or in immunocompetent SCC cases [7]. This could explain the lower seroprevalence of MCV T-Ag observed among SCC cases compared to that of viral capsid antigens, as well as the lower seroprevalence of MCV T-Ag in SCC cases compared to that reported among MCC cases [16].

The lack of an association between seropositivity to MCV T-Ag and SCC, and low viral DNA load in SCC tumors, suggest that MCV is not directly involved in the development of SCC. Further, the ratio of N-terminus to C-terminus of MCV T-Ag was close to 1.0 in MCV DNA-positive SCC (Fig. 1), indicating a lack of the tumor-promoting, signature T-Ag mutations described for MCC. Despite the lack of evidence for a causal role of MCV in SCC, an indirect role of MCV cannot be ruled out. In the present study, a four-fold association was observed between the presence of MCV DNA in eyebrow hairs and MCV DNA-positive SCC. This strong association, consistent with our previous report of a positive association between MCV seropositivity to capsid antigen and MCV DNA-positive SCC [11], suggests MCV may be a marker of an unknown immunological factor associated with SCC, acting either synergistically with or independently of MCV infection. However, in this retrospective case–control study, it is not possible to distinguish between immunological factors that predispose to SCC versus those that result from the presence of the cancer itself. A prospective study examining MCV infection in multiple biospecimens prior to the onset of SCC and markers of immune function is needed to elucidate the precise role of MCV in SCC.

## Abbreviations

MCC: Merkel cell carcinoma
MCV: Merkel cell polyomavirus
SCC: Squamous cell carcinoma
VP1: Capsid antigen
T-Ag: T-antigen
MFI: Median fluorescence intensity
PCR: Polymerase chain reaction
OR: Odds ratios
CI: Confidence intervals
